# Profiling of skeletal muscle tissue for long non-coding RNAs related to muscle metabolism in the QingYu pig at the growth inflection point

**DOI:** 10.5713/ajas.20.0429

**Published:** 2020-10-13

**Authors:** Jia Luo, Linyuan Shen, Mailin Gan, Anan Jiang, Lei Chen, Jideng Ma, Long Jin, Yihui Liu, Guoqing Tang, Yanzhi Jiang, Mingzhou Li, Xuewei Li, Shunhua Zhang, Li Zhu

**Affiliations:** 1 College of Animal Science and Technology, Sichuan Agricultural University, Chengdu 611130, China; 2 College of Animal Science and Technology, Southwest University, Chongqing 400715, China; 3 Farm Animal Genetic Resource Exploration and Innovation Key Laboratory of Sichuan, Chengdu 611130, China; 4 Sichuan Province General Station of Animal Husbandry, Chengdu 610041, China; 5 College of Life Science, Sichuan Agricultural University, Ya’an 625014, China

**Keywords:** lncRNA, Growth Curve, Inflection Point, Muscle, Metabolism, QingYu Pig

## Abstract

**Objective:**

Investigation of muscle growth at different developmental stages is an appropriate strategy for studying the mechanisms underlying muscle development and differences in phenotypes. In particular, the muscle development mechanisms and the difference between the fastest and slowest growth rates.

**Methods:**

In this study, we used a growth curve model to fit the growth inflection point (IP) of QingYu pigs and compared differences in the long non-coding RNA (lncRNA) transcriptome of muscle both at the growth IP and plateau phase (PP).

**Results:**

The growth curve of the QingYu pig had a good fit (R^2^ = 0.974) relative to a typical S-curve and reached the IP at day 177.96. At the PP, marbling, intramuscular fat, and monounsaturated fatty acids had increased significantly and the percentage of lean muscle and polyunsaturated fatty acids had decreased. A total of 1,199 mRNAs and 62 lncRNAs were differentially expressed at the IP compared with the PP. Additional to gene ontology and Kyoto encyclopedia of genes and genomes pathway analyses, these differentially expressed protein coding genes were principally related to muscle growth and lipid metabolism.

**Conclusion:**

Our results suggest that the identified differentially expressed lncRNAs, could play roles in muscle growth, fat deposition and regulation of fatty acid composition at the IP and PP.

## INTRODUCTION

Myogenesis is a complex process of the formation of muscle fibers. The formation of new multinucleated muscle fibers from mononucleated precursor cells termed myoblasts, is exclusively a prenatal process that determines the characteristics of muscles, such as numbers of fiber, possibly related to muscle strength and function [1]. In pigs, muscle growth is predominantly determined during prenatal skeletal muscle development [2]. Pigs have been genetically selected for efficient muscle growth over many previous decades, which means they are an interesting model for the study of myogenesis [3]. The classical growth development model of the pig is a sigmoidal curve. After birth, the growth rate increasing slowly until the maximum growth rate is reached at the inflection point (IP), then decreases asymptotically, reducing to a minimum at the plateau [4]. Improving growth rate and muscularity has been the primary focus of pig breeders during past decades. Therefore, muscle phenotypic difference between the maximum growth rate point (at the IP) and the minimum growth rate point (at the plateau phase, PP) represent a potentially good model for studying the molecular mechanism of muscle development. Study of the underlying complex molecular mechanisms of muscle development is beneficial for the genetic improvement of meat quality and lean meat percentage. Moreover, since pigs have similar physiological, pathological and genomic characteristics to humans, understanding the molecular mechanisms of myogenesis has important implications for understanding muscle development and muscle regeneration.

The majority of long non-coding RNAs (lncRNAs) are expressed at particular stages of biological development in a specific manner for different cells or tissues. Emerging research has shown that lncRNAs participate in the development of skeletal muscle [5], lipid deposition and adipogenesis [6]. To identify genes that affect muscle growth rates, various studies have focused on muscle transcriptome diversity at different developmental stages in the pig [7]. So far, thousands of lncRNAs have detected related to muscle growth and development. For example, Zhao et al [8] identified more than 570 lncRNAs by systematically analyzing lncRNA expression in skeletal muscle at different times. However, no studies were focused on lncRNAs at the extremes of growth rate defined by a growth curve, such as the growth IP or the PP.

The QingYu pig is an indigenous breed from Southwestern China that is medium-sized and famous for its excellent meat quality attributes. Furthermore, this breed is classified as a meat and fat dual-purpose breed. To ascertain the growth characteristics of the QingYu pig, its growth curve was modeled. And then the maximum grow rate occurring at IP was estimated. A comprehensive survey of global gene expression changes in the longissimus dorsi muscle was subsequently conducted at IP and PP. The present study was to identify differentially expressed lncRNAs linked to phenotypic differentiation (e.g. growth rate, meat quality) between the maximum growth rate point (at IP) and the minimum growth rate point (at PP). This study will serve as a valuable resource to study muscle development and it is beneficial to the breeding of animal growth and development.

## MATERIALS AND METHODS

All animal procedures were conducted in accordance with institutional guidelines for the care and use of laboratory animals and were approved by the Animal Care and Ethics Committee of Sichuan Agricultural University, Sichuan, China, under Permit number DKY-B20161705.

### Animal and sample preparation

Animal experiments were organized by Sichuan Agricultural University and conducted at BaShan Animal Husbandry Limited Technology Co., Ltd. Growth development data of 126 captive QingYu pigs (56 males and 60 females) at 18 time points were collected from birth to 400 d in an unselected, random mating population. Body weight and other measurements were collected from two batches of the pigs. The two batches have uniform environmental conditions except for a difference in age of birth of 20 days. Growth weight was measured then a growth curve fitted using three non-linear models. All pigs were housed in individual pens (2 m^2^) located in the same room and fed twice daily using the same diet, with *ad libitum* access to water, using nipple drinkers. Experimental diets, based on corn and soybean meal, were formulated with crude protein, trace mineral and vitamin concentrations that met or exceeded the National Research Council (NRC, 1998) recommendations for different growth phases. At slaughter age (IP, 178 days old and PP, 395 days old), mean body weights of the pigs were 66.98±6.6 kg and 145.9±9.5 kg, respectively. After slaughter, the longissimus dorsi muscle was sampled into 2 mL tubes within 30 min, immediately frozen in liquid nitrogen then transferred to a freezer at −80°C for long-term preservation and additional total RNA extraction, if required.

### Growth curve models

There are three nonlinear models involved in our study. The first is the logistic growth curve model, the equation is W_t_ = A/(1+B*e*^−^*^kt^*). The second is the Gompertz growth curve model, defined by the equation. The third is the Von Bertalanffy growth curve model, which is defined by the equation W_t_ = A×(1−B*e*^−^*^kt^*)^3^. When fitting a curve by these models, lnB/k, lnB/k, and lnB/k represent the inflection age; A/2, A/e, and 8A/27 represent the inflection weight; kw/2, kw, and 3 kw/2 represent the maximum daily gain. In the above equations, W_t_ represents the time point where the weight was recorded, A represents the maximum size, k may be interpreted as the inherent relative growth rate at the start, and B is the growth curve line constant.

Goodness-of-fit (R^2^) was used to judge the merits of the fitting model, the equation is ${\text{R}}^{2}=1-\frac{\text{RSE}}{\text{RST}}$, where R^2^ represents the goodness-of-fit, RSE represents the residual sum of squares, and RST represents the sum of squares of deviations.

### Carcass and meat quality measurements

After evisceration, the left half carcass was weighed, and the dressing percentage calculated. The right half of the carcass was used to assess morphometric parameters. Specifically, carcass length was measured using a flexible tape. The loin eye area was also measured at the level of the 6th to 7th rib. The dorsal fat thickness was measured with a flexible tape at the level of the first rib, the last rib, and in the region where the dorsal fat was the thickest. The mean of these three measurements was used for the comparison of dorsal fat values.

Meat quality traits including muscle pH, meat color, drip loss, cooking loss and Warner-Bratzler shear force (WBS) were measured at both 45 min post-mortem. Marbling scores were evaluated 24 h post-mortem using a published visual standard (NPPC, 1991). Muscle pH was measured at approximately 1 cm below the cutting surface of longissimus dorsi (3rd to 4th rib) using a pH-star meter (SFK Inc., Berlin, Germany). Meat lightness (CIE L*) was also objectively measured in the cutting surface of longissimus dorsi between 5th and 6th rib, using the Model CR-300 Minolta Chroma Meter (Minolta, Ramsey, NJ, USA) fitted with a 50-mm-diameter aperture, using a D65 illuminant. Drip loss was determined by weighing sliced meat stored at 4°C after 24 h post-mortem, and calculated as a percentage original weight of the sliced meat. To determine cooking loss, a 2.5 cm thick (approximately 100 g) section of loin sample was cooked to an internal temperature of 70°C in a steamer. Cooking loss was determined by calculating the weight difference between cooked and uncooked samples. The WBS was determined using a Texture Analyzer (TA.XT. Plus, Stable Micro Systems, Godalming, UK) equipped with a Warner-Bratzler shearing device. For determination of intramuscular fat content, 50 g samples of loin meat were collected, and the IMF was analyzed using the Soxhlet method.

### Fatty acid composition

For fatty acid composition, lipids were extracted with chloroform and methanol. For lipid hydrolysis, an aliquot of lipid extract (30 mg) and 3 mL of 4% H_2_SO_4_ in methanol were combined in a screw-capped test tube. The test tube was placed in boiling water (100°C) for 20 min and subsequently cooled at room temperature. The resulting free fatty acids were methylated with 1 mL of 14% boron trifluoride in methanol at room temperature for 30 min. Water (1 mL) and hexane (5 mL) were added. Samples were vortexed and centrifuged at 500×g for 10 min. The upper organic solvent layer was used to determine fatty acids composition. Fatty acid methyl esters were analyzed on a gas chromatograph (Agilent Technologies 6890N, Santa Clara, CA, USA) equipped with an on-column injection port and flame-ionization detector. A Silica capillary column (Omegawax 320, 30 m×0.32 mm×0.25 μm film; Supelco, Bellefonte, PA, USA) was used for the separation of the fatty acid methyl esters. The gas chromatography oven temperature was 140°C, and increased at a rate of 2°C/min to a final temperature of 230°C. The temperatures of injector port and detector temperatures were set at 240°C and 250°C, respectively. Fatty acid methyl ester (1 μL) was injected onto the split injection port (100:1 split ratio). The flow rate for He carrier gas was 50 mL/min. Each fatty acid was identified by its retention time.

### Isolation of total RNA and quality control

Longissimus dorsi muscles at IP and PP from three female pigs were harvested for total RNA isolation. A mirVana RNA isolation kit (#AM1561, Ambion, Austin, TX, USA) was used to isolate total RNA, in accordance with the manufacturer’s instructions. Isolated total RNA from each sample was preserved at −80°C. A NanoDrop 2000 spectrophotometer (Thermo Scientific, Waltham, MA, USA) was used to determine RNA concentration from the optical density (OD)-260 nm/OD-280 nm absorption ratio, which was controlled in the range of 1.9 to 2.1. A Bioanalyzer 2100 (Agilent, USA) was used to evaluate the quality of total RNA (RNA integrity number ≥7 and 28S/18S ≥0.7). RNA integrity number was larger than 8.0 in all samples. RNase-free DNase I (Ambion Inc., USA) was used to eliminate potential genomic DNA contamination.

### RNA-seq, data processing and gene annotation

Total RNA was extracted from longissimus dorsi of IP and PP using TRIZOL (Invitrogen, Carlsbad, CA, USA), and further purified with RNeasy column (Qiagen, Valencia, CA, USA) according to the manufacturer’s protocol. Three biological replicates in each group were used to construct transcriptome Library. Approximately 3 μg of total RNA from each sample were used for the construction of cDNA libraries (including IP1, IP2 and IP3; PP1, PP2, and PP3), in accordance with the Illumina TruSeq RNA sample preparation guide. The process included: i) separation, enrichment and purification of mRNA using oligo (dT) magnetic beads; ii) enzymatic fragmentation of RNA; iii) synthesis of cDNA; iv) sequencing adapter ligation; and v) polymerase chain reaction (PCR) amplification. An Agilent DNA 1000 kit was used to determine the size and purity of cDNA libraries on an Agilent 2100 Bioanalyzer (Agilent technologies, USA). An ABI StepOnePlus qRT-PCR system was used to accurately determine the effective concentration of cDNA libraries (>2 nmol/L), thus ensuring their quality. An Illumina HiSeq 2500 platform was used for paired-end sequencing of cDNA libraries from which raw reads were obtained. The raw reads were transferred from original image data by base calling. Quality control and reads statistics were determined by FASTQC v0.11.2. After discarding the reads containing adapter, reads containing over 10% poly-Ns, and reads of low quality (>50% of bases with Phred scores <5), the remaining clean reads were aligned to the reference *S. scrofa* genome (10.2) using TopHat v2.0.9, package, and parameters were set as default.

The RNA sequencing data were uploaded to NCBI’s Gene Expression Omnibus, and can be found under Accession Number GSE139322 https://www.ncbi.nlm.nih.gov/geo/query/acc.cgi?acc=GSE139322.

### Identification of lncRNAs and mRNAs

All the downstream analyses were based on high quality clean data. The mapped reads from each library were assembled with Cufflinks (version 2.1.1) to construct and identify mRNA transcripts. Next, the data analysis was performed by filtering the assembled novel transcripts from the different libraries to obtain putative lncRNAs following the steps in the pipeline as follows. First, transcripts that were shorter than 200 nt in length, containing fewer than 1 exon and fewer than three reads were excluded. Next, using the coding-non-coding index (CNCI) and coding potential calculator (CPC) to evaluate the coding potential of the filtered transcripts. A transcript with a CNCI value lower than 0 and a CPC value lower than −1 was taken to be an lncRNA. The expression levels of mRNAs and lncRNAs were quantified using a TPM algorithm [9].

### Analysis of differentially expressed genes

Three biological replicates were utilized in this experiment. Differentially expressed lncRNAs and mRNAs were identified based on a negative binomial distribution using the Noiseq package [10]. Differentially expressed mRNAs and lncRNAs were filtrated by Cuffdiff software with parameters of p value <0.05 and |log2 (fold change)| ≥1.

### Neighboring genes prediction and functional analysis of differentially expressed lncRNAs

LncRNAs can cis-regulate neighboring genes. For expression pattern analysis, a Pearson correlation coefficient (PCC) was calculated for expression values of each lncRNA and mRNA. The PCCs of expression levels of differentially expressed lncRNAs and mRNAs were calculated. |PCC|>0.8 and p value <0.05 were the threshold according to which co-expressed lncRNA-mRNA were selected. Genes transcribed within a 100-kb window upstream or downstream of lncRNAs were considered cis neighboring gene s if the |PCC| of lncRNA-mRNA >0.9. Gene ontology annotation and Kyoto encyclopedia of genes and genomes (KEGG) [11] pathway enrichment analysis were conducted for those neighboring genes to investigate the biological processes and signaling pathways with which the lncRNAs were principally involved and their functions.

### Quantitative real-time polymerase chain reaction verification

The relative expression levels of selected genes were quantified using real-time RT-PCR analysis same as our previous research. Briefly, total RNA was extracted using Trizol reagent (Invitrogen Corp, USA), accordance with the manufacturer’s instructions. Reverse transcription was performed using oligo (dT) random 6-mers primers provided in the PrimeScript RT Master Mix kit (TaKaRa, Dalian, China). Quantitative PCR was conducted using a SYBR Premix Ex Taq kit (TaKaRa, China) with using a CFX96 real-time PCR detection system (Bio-Rad, Richmond, CA, USA). All measurements included a negative control (no cDNA template) and each RNA sample was analyzed in triplicate. Relative expression levels of the target mRNAs and lncRNAs were calculated using the 2^−ΔΔ^Ct method against the endogenous control glyceraldehyde-3-phosphate dehydrogenase.

### Statistical analysis

All data are presented as means±standard deviations. The significance of comparisons between the IP and PP were calculated using a Student’s t-test. Statistical significance is defined when p values are less than 0.05. Benjamin-corrected modified Fisher’s exact test was used to calculate the p values.

## RESULTS

### Growth curve of the QingYu pig

**Figure 1:**
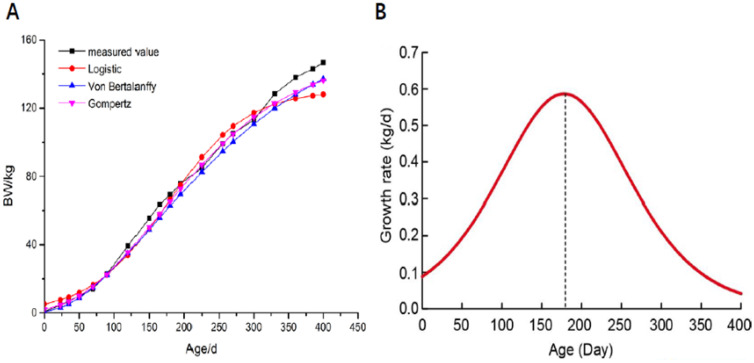
The fitting growth curve of QingYu pig. (A) Growth curve of QingYu pig, four curves include one measured value and three curves fitted by three different models (Logistic, Gompertz and Von Bertalanffy). (B) Growth rates of QingYu pig.

The growth curve and growth rates of the QingYu pig are displayed in Figure 1. The QingYu pig growth curve fitted well to a typical S-curve. The relative growth intensity of younger pigs was greater than that of older pigs, and decreasing gradually with age. As shown in Table 1 and Figure 1, all fitted lines were closed to the observed experimental values, the models able to be ranked according to their R^2^ values as logistic (0.974). The growth curve suggested that QingYu pigs reached their maximum growth rate at IP (at day 177.96) when the mean body weight was 65.20 kg, then the growth rate gradually declined. Based on the growth curve of QingYu pigs, the longissimus dorsi muscle was harvested at the IP (day 178, IP) and growth plateau (day 395, PP) for phenotypic and transcriptome analysis in order to reveal potential molecular regulation mechanisms of muscle growth and metabolism.

### Phenotypic differences of muscle at the inflection point and plateau phase

Carcass and meat quality is influenced by the age [12,13]. To determine differences at different developmental stages, the carcass and meat quality characteristics of the QingYu pig at IP and PP were compared based on the growth curve of QingYu pigs (Table 2). The dressing percentage, back fat, cooking loss, marbling and intramuscular fat all increased significantly (p<0.05) at the PP. Fat development within muscle is not late maturing, however, marbling and intramuscular fat are known as a late maturing trait. Moreover, the expression of marbling is due to maintained fat synthesis in combination with declining muscle growth as animals get older [14]. Moreover, lean percentage, drip loss and pH decreased significantly (p<0.05). These findings demonstrate that fat deposition was enhanced in later feeding.

**Figure 2:**
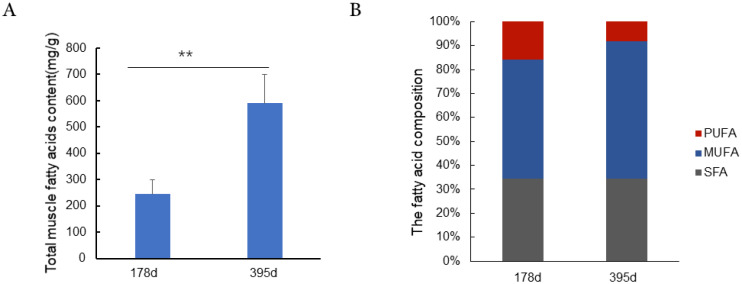
The fatty acid composition of QingYu pig at IP and PP, 5 pigs were used for the analysis. (A) Total muscle fatty acids content (mg/g). (B) Fatty acid composition. IP, the growth inflection point (day 178); PP, the plateau phase (day 395); PUFA, polyunsaturated fatty acid; MUFA, monounsaturated fatty acid; SFA, saturated fatty acid. ** p<0.01.

Fatty acids are the principal substrate and primary fuel source of lipid metabolism, also closely associated with adipocyte development [15]. Furthermore, fatty acid content is an important factor that affects the flavor of meat. Fatty acids are divided into three types according to the degree of saturation: saturated fatty acids (SFA), monounsaturated fatty acids (MUFA), and polyunsaturated fatty acids (PUFA). Cameron et al. found the longissimus dorsi muscle in Duroc pigs had higher SFA and MUFA content, with lower concentrations of PUFA than in Landrace pigs. However, the fatty acid content and composition in different growth phases is unclear. Therefore, we analyzed the fatty acid content and composition at the IP and PP (Figure 2). Our results indicated that muscles at the PP exhibited significantly greater total fatty acid and MUFA content than at the IP (p<0.001). PUFA concentration at the PP was significantly lower than that of the IP (p<0.05). Oleic acid (C18:1) content was 110.07 mg/g at the IP vs 302.44 mg/g at the PP. In addition, both C18:2 (linoleic acid, 28.23 vs 36.65 mg/g) and C18:3 (linolenic acid, 0.57 vs 0.69 mg/g) content was higher at the PP.

### Comparison of mRNA and lncRNA characteristics

Based on the growth curve of QingYu pigs, the longissimus dorsi muscle was harvested at the IP and PP for transcriptome analysis. To comprehensively understand the molecular regulation mechanisms of muscle growth based on the growth curve of the QingYu pig, total RNA of muscle harvested at the IP and PP was isolated and sequenced using an Illumina sequencing platform. The Q30 was not less than 91.78%. After filtering, approximately 89,680,136 of clean reads per library were typically obtained. More than 94% were mapped to the pig reference genome (Sscrofa 11.1), among them, 54.06% to 64.33% had a unique genomic position (Supplementary Table S1). A total of 22,896 transcripts were obtained, including 22,672 that were filtered according to transcriptome assembly and reconstruction. Together, these results suggest that the RNA-sequencing data were credible.

In order to further explore the characteristics of lncRNAs identified in this study, a bioinformatics analysis was performed, from which 2,192 lncRNAs and 61,379 mRNAs were identified. As shown in Supplementary Figure S1, lncRNAs and mRNAs exhibited significant differences in expression level, sequence length and exon number. FPKM values for the transcripts indicated that mRNA expression levels were relatively higher than those of lncRNAs (Supplementary Figure S1A), consistent with previous research results [16]. The majority of lncRNAs possessed one exons, significantly fewer than those of protein-coding genes. Overall, the distribution of lncRNA and mRNA lengths were consistent, and the proportion of relatively long mRNA transcripts higher than those of lncRNAs (Supplementary Figure S1B). Mean lncRNA and mRNA length was 2,217 and 2,329 nt, respectively. mRNA transcripts were much longer than lncRNAs. The length of the majority (53.99%) of lncRNAs was between 200 and 1,000 bp, compared to most (95.30%) mRNAs being between 600 bp and 3,000 bp. A comparison of mRNA and lncRNA characteristics revealed that 83.71% of the lncRNA transcripts contained 1 or 2 exons, and that 89.87% of the mRNA transcripts had ≥3 exons (Supplementary Figure S1C). In addition, the number of exons in lncRNAs in muscle tissue was consistent with the number in other pig tissues.

### Differential expression of genes related to muscle growth and fatty acid composition

**Figure 3:**
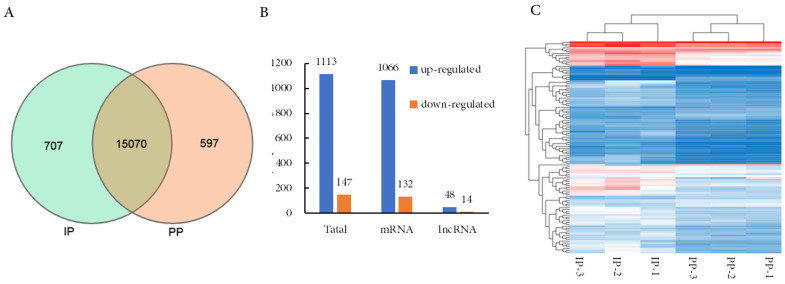
The expression levels of mRNA between IP and PP. (A) The Ven diagram of mRNAs that identified in two development phases. (B) The number of differentially expressed mRNA between IP and PP. (C) The hierarchical cluster analysis for the differentially expressed mRNA. IP, the growth inflection point (day 178); PP, the plateau phase (day 395).

A Venn Diagram indicated that a total of 707 mRNAs were expressed specifically at the IP and 597 mRNAs specifically at the PP of the QingYu pig (Figure 3A). A total of 1,260 differential genes, include 62 differentially expressed lncRNAs (48 up-regulated and 14 down-regulated) and 1,198 differentially expressed mRNAs (1,066 up-regulated and 132 down-regulated) were identified (Figure 3B). Heatmaps of the differentially expressed genes at the IP compared with the PP suggest that they could be distinguished by clustering, reflecting the reliability of biological duplication in this study (Figure 3C).

**Figure 4:**
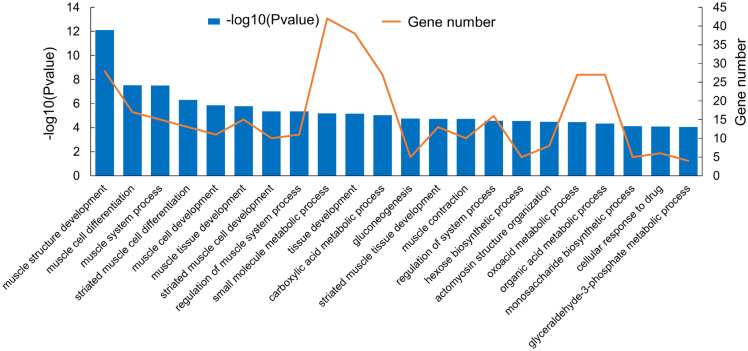
The functional enrichment analysis of differentially expressed mRNA in different gene ontology terms.

**Figure 5:**
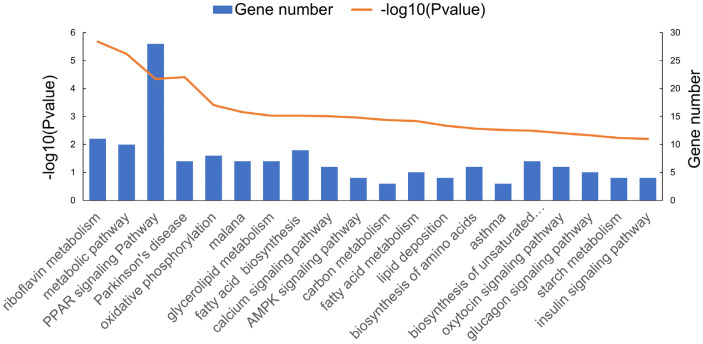
The functional enrichment analysis of differentially expressed mRNA in different Kyoto encyclopedia of genes and genomes pathway.

In order to elucidate the biological functions of differentially expressed mRNAs and reveal the biological differences between muscle tissues at the IP and PP, functional enrichment analysis (including GO and KEGG Pathway analysis) was conducted on the differentially expressed mRNAs. GO enrichment analysis results demonstrated that many of the significantly enriched GO terms were closely associated with muscle growth and development. As shown in Figure 4, in the biological process category, many genes (≥10) were enriched in “muscle structure development”, “muscle cell differentiation”, “muscle system process”, “muscle cell development”, and “muscle tissue development”. To further analyze the development-specific function of differentially expressed transcripts, a KEGG pathway enrichment analysis of differentially expressed mRNAs at the IP compared with those at the PP was conducted. As shown in Figure 5, many genes were significantly enriched in signaling pathways closely related to muscle growth and lipid metabolism, including “peroxisome proliferator activated receptor (PPAR) signaling pathway”, “AMP-activated protein kinase signaling pathway”, “oxidative phosphorylation”, “fatty acid metabolism”, and “fatty acid biosynthesis”, etc.

### Neighboring genes and functional analysis of differentially expressed lncRNAs

**Figure 6:**
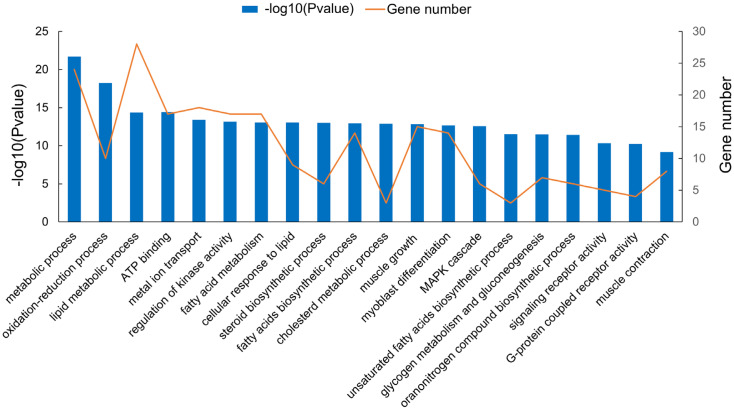
The functional enrichment analysis of cis-target genes by differentially expressed long non-coding RNAs. in different gene ontology terms.

**Figure 7:**
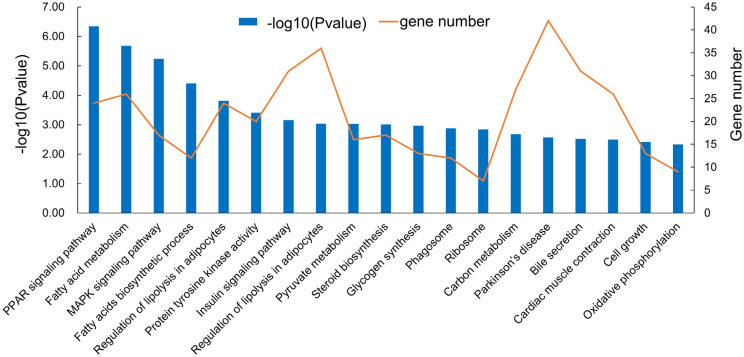
The functional enrichment analysis of cis-target genes by differentially expressed long non-coding RNAs in different Kyoto encyclopedia of genes and genomes pathway.

A total of 62 differentially expressed lncRNAs (48 up-regulated and 14 down-regulated) were identified. To investigate the function of lncRNAs, we predicted the potential cis targets of lncRNAs, using 100 kb windows from the lncRNA genes. Fifty six differentially expressed protein-coding genes were found close to 62 differentially-expressed lncRNA genes. GO term enrichment analysis of differentially expressed lncRNA cis-regulated predicted neighboring genes were mainly enriched in ‘lipid metabolic process’, ‘lipid biosynthetic process’, ‘fatty acids biosynthetic process’, ‘muscle growth’, and ‘myoblast differentiation’ (Figure 6). KEGG terms that were closely associated were: ‘mitogen-activated protein kinase (MAPK) signaling pathway’, ‘PPAR signaling pathway’, ‘regulation of lipolysis in adipocytes’, ‘fatty acids biosynthetic process’, and ‘fatty acid metabolism’, etc. (Figure 7). The MAPK and PPAR signaling pathways were key to the regulation of skeletal muscle development, adipocyte differentiation and lipid accumulation.

### Quantitative real-time polymerase chain verification

**Figure 8:**
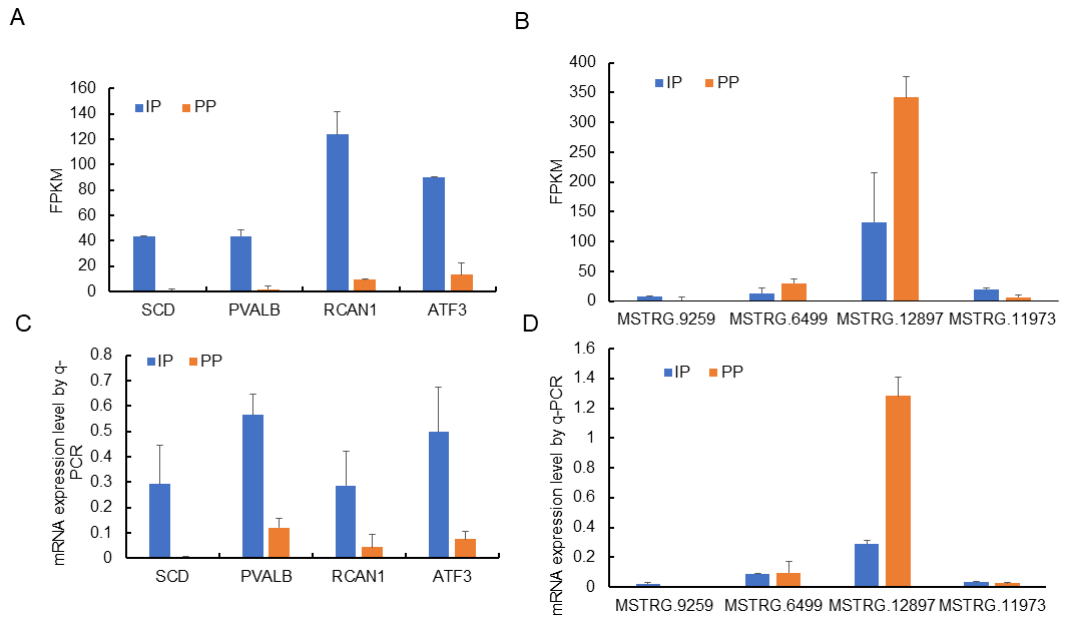
qRT-PCR validation of the differentially expressed genes. The differential expression of genes in muscle between IP and PP was validated by qRT-PCR, reference genes was glyceraldehyde-3-phosphate dehydrogenase. qRT-PCR, quantitative real-time polymerase chain reaction; IP, the growth inflection point (day 178); PP, the plateau phase (day 395).

To validate the reliability of RNA-seq results, differentially expressed genes (four lncRNAs and four mRNAs) were randomly selected for further qRT-PCR verification. qRT-PCR is considered the gold standard for quantitative analysis of gene expression. The results indicated that stearoyl-CoA desaturase (SCD), parvalbumin (PVALB), regulator of calcineurin 1 (RCAN1), activating transcription factor 3 (ATF3), MSTRG.6499, and MSTRG.12897 were highly expressed in IP, while MSTRG.9259, MSTRG.11973 were highly expressed in PP (Figure 8). These data were consistent with the sequencing results, verifying the reliability of the sequencing results.

## DISCUSSION

According to the growth curve, QingYu pigs reached their IP at day 177.96. QingYu pigs reached their growth IP earlier and have heavier body weight than another Chinese native breeds, such as Liangshan pigs, that reach their growth IP at day 193.40 with a mean body weight of 62.5 kg [17]. However, QingYu pigs reached their growth IP later and have a lower body weight than Durocs, reaching a growth IP at 163.6 days at 134.6 kg on average [18]. The maximum daily gain of QingYu pigs at IP was 586.82 g/d, lower than the Large White at 659.08 g/d considerably [19]. However, similar to other reports of native Chinese pigs, the weight at the IP of the Liangshan pig is 62.5 kg with a maximal daily gain of 455.43 g and Chenghua pig is 77.73 kg with a maximal daily gain of 430 g [17]. These results suggest that QingYu pig has a lower growth rate than foreign breeds, related to the lack of long-term artificial selection for growth rate.

The marbling and intramuscular fat are increased significantly at the PP (p<0.05). The marbling and intramuscular fat are known as a late maturing trait and the increased marbling expression is due to the enhanced fat synthesis as animals get older. These findings demonstrate that fat deposition was enhanced in later feeding. Fatty acid composition not only affects the flavor of meat products, but is also closely related to human health. According to previous reports, PUFAs are easier to oxidize and their presence is associated with an off-flavor [20]. Meat with higher PUFA content may have a worse flavor and is hard to store. Oleic acid (C18:1) content was 110.07 mg/g at the IP vs 302.44 mg/g at the PP. Reports have demonstrated that increasing C18:1 concentration pre-accelerates adipocyte differentiation, causing an increase in adipose tissues mass [21]. In addition, both C18:2 (linoleic acid, 28.23 vs 36.65 mg/g) and C18:3 (linolenic acid, 0.57 vs 0.69 mg/g) content was higher at the PP. Linoleic and linolenic acids are essential fatty acids in humans and the substrate for the synthesis of a variety of long-chain PUFAs such as arachidonic acid, docosapentaenoic acid and docosahexaenoic acid [22]. Their derivatives are involved in many pathways of metabolic regulation and structural formation, playing a crucial role in the growth and development of the brain [23]. These results suggest that the QingYu pig had superior muscle fatty acid composition at the PP.

Many differentially expressed genes were significantly enriched in signaling pathways closely related to muscle growth and lipid metabolism, including “muscle structure development”, “muscle cell differentiation”, “muscle cell development” and “PPAR signaling pathway”, “AMPK signaling pathway”, “oxidative phosphorylation”, “fatty acid metabolism” and “fatty acid biosynthesis”, etc. The AMPK signaling pathway, an important signaling system that mediates the response of cells to external stimuli, was key to regulating myoblast differentiation and fatty acid oxidation. The PPAR signaling pathway is associated with adipocyte differentiation and lipid metabolism [24]. In addition, MUFA synthesis increased with weight gain and muscle development. SCD1 is a key enzyme that converts SFAs to MUFAs and related to the biosynthesis of C18:1 and C16:1. Other important fatty acid desaturases, such as SCD2, fatty acid desaturase (FADS), were also implicated. In this study, the expression of SCD1, FADS1, and FADS2 was higher at the PP. SCD1 exhibited the biggest difference between the IP and PP. As Wang et al [25] found that genes involved in fatty acid conversion catalyzed the conversion of SFA to MUFA. However, we found SFAs were little changed between the IP and PP in QingYu pigs, whilst MUFAs increased. This finding suggests that gene expression and the biological process of fatty acid composition and metabolism in muscle may involve in other metabolic pathways.

In this research, we further predicted the potential cis targets of lncRNAs. Fifty-six differentially expressed protein-coding genes were found close to 62 differentially-expressed lncRNA genes. GO and KEGG analysis found that differentially expressed lncRNA cis-regulated predicted neighboring genes were mainly enriched in fat metabolism and muscle growth related pathways, such as ‘lipid metabolic process’, ‘muscle growth’, ‘MAPK signaling pathway’ and ‘PPAR signaling pathway’, etc. Among them, the MAPK and PPAR signaling pathways were key to the regulation of skeletal muscle development, adipocyte differentiation and lipid accumulation. Emerging research has demonstrated that lncRNAs participate in the development of skeletal muscle and fat deposition and metabolism in livestock and thousands of lncRNAs have been identified. For example, 55 lncRNAs that were expressed differentially in high intramuscular fat liver compared with low intramuscular fat liver in pigs [26]. Linc-RAM enhances myogenic differentiation by interacting with MyoD, reveal the functional role of a Linc-RAM as a regulatory lncRNA required for tissues-specific chromatin remodeling and gene expression [27]. Linc-YY1 promotes myogenic differentiation and muscle regeneration through an interaction with the transcription factor YY1 [28]. Additionally, several lncRNAs and protein coding genes associated with muscle development were screened in sheep using RNA-sequencing. All studies described above indicated that lncRNAs play an important role in muscle growth, fat deposition and regulation of lipid metabolism at the IP and PP.

## CONCLUSION

In this study, we fitted the growth curve and evaluated the growth rate of QingYu pigs. The pigs reached IP at day 177.96. Marbling, intramuscular fat and presence of MUFA increased significantly at PP. The longissimus dorsi muscle was selected for transcriptome analysis at IP and PP. RNA-seq technology and bioinformatics analysis were utilized to identify differentially expressed lncRNAs and mRNAs of muscle tissue between the IP and PP to explore the molecular mechanisms of phenotypic differentiation. Results suggest that some of the protein-coding genes that they identified as differentially expressed are involved in processes and pathways related to muscle development, fatty acid synthesis and metabolism. This study provides a useful reference for better understanding the molecular mechanisms of animal growth and lipid metabolism based on a growth curve.

## Supplementary Information



## Figures and Tables

**Table 1:** The estimated value of growth curve and fitting degree of different models

Models	k	a	b	R^2^	GTPD (d)	GTPW (kg)	TPGR (g)
Logistic	135.63	22.075	0.017	0.999	182.03	67.72	576.4
Von Bertalanffy	198.903	0.826	0.005	0.998	181.49	58.93	442
Gompertz	165.829	4.141	0.008	0.996	185.6	61.01	488.08

k, the inherent relative growth rate at the start; a, the maximum size; b, the growth curve line constant; R^2^, the goodness-of-fit; GTPD, growth turning point day; GTPW, growth turning point weight; TPGR, the biggest growth rate.

**Table 2:** Carcass, meat quality characteristics of QingYu pig at the inflection point (IP) and plateau phase (PP)

Age/d	IP	PP	p-value
Slaughter weight (kg)	66.98±6.6	145.9±9.5	<0.001
Carcass weight (kg)	44.64±3.99	112.97±8.55	<0.001
Dressing percentage (%)	66.86±5.03	77.73±1.92	<0.05
Back fat (mm)	33.28±5.4	52.29±9.03	<0.05
Eye muscle (cm^2^)	17.84±3.67	19.46±5.26	>0.05
Lean percentage (%)	43.32±2.94	35.54±2.00	<0.05
Leg hip ratio (%)	25.64±0.76	25.73±2.02	>0.05
Marbling	2.63±0.41	2.75±0.28	<0.05
Drip loss (%)	1.90±0.50	1.75±0.28	<0.05
Cooking loss (%)	66.03±2.29	70.48±1.49	<0.05
pH	6.80±0.31	6.37±0.32	<0.05
Muscle color	43.65±3.25	44.43±1.30	>0.05
Shear force	6.05±2.67	6.07±1.47	>0.05
Intramuscular fat	2.48±0.30	6.52±2.77	<0.01

The significance of comparisons between the inflection point (IP) and plateau phase (PP) were calculated using a Student’s t-test.

p<0.05 was considered statistically significant, p<0.001 was considered statistically extremely significant, p>0.05 was considered statistically no significant.
