# Supplementary material for: Profiling of skeletal muscle tissue for long non-coding RNAs related to muscle metabolism in the QingYu pig at the growth inflection point
Source: Anim Biosci. 2020 Oct 13;34(8):1309–20. doi: 10.5713/ajas.20.0429 (PMC8255898; doi:10.5713/ajas.20.0429)
Supplement: Supplementary file 1 [file ajas-20-0429-suppl.pdf]

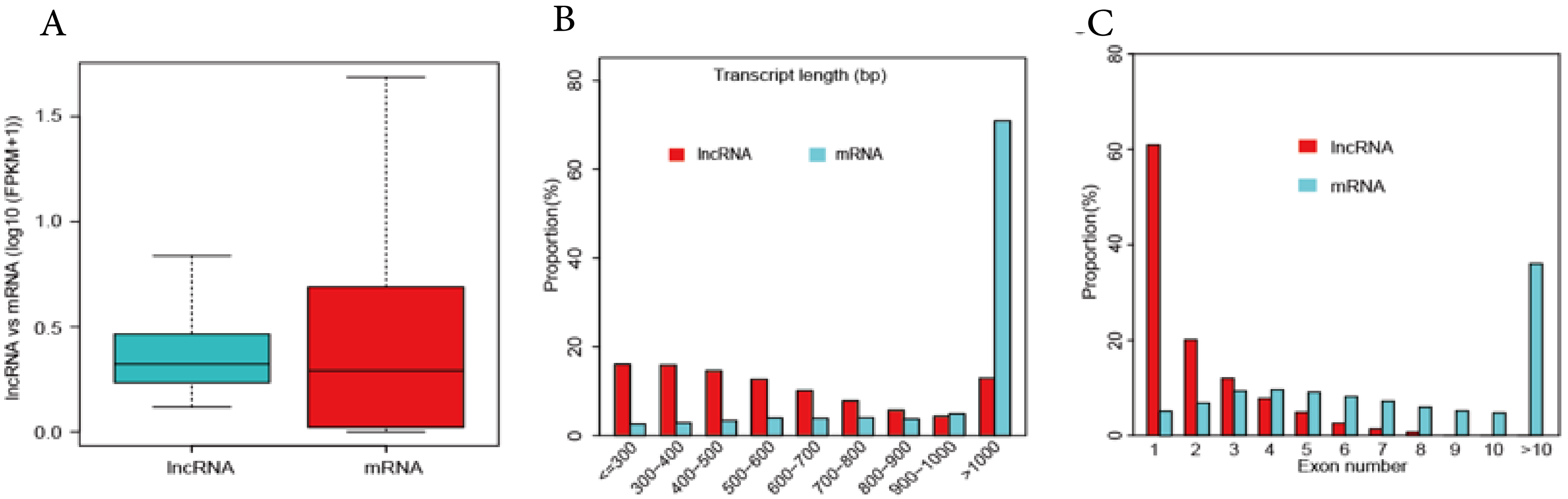

Fig. S1. Comparison of genomic architecture between mRNAs and lncRNAs. (A) Box plot indicating expression level of total mRNAs and lncRNAs by log10(TPM + 1). (B) Distribution of transcript lengths of mRNAs and lncRNAs. The x axis represents the length of transcripts, and the y axis represents composing proportion. (C) Distribution of the number of exons in mRNAs and lncRNAs. Single-exon lncRNAs were filtered out from the pig genome.

Table S1 Summary of clean reads after Filtering and Mapping to the reference genome

Sample	Total Clean R	Mapped reads	Percentage of	Percentage of	Clean Data Q	Clean Data Q	GC Content (%)
PP_1	89,134,050	83,988,897	94.23%	54.06%	96.55	91.88	54.45
PP_2	89,761,138	84,554,534	94.20%	59.20%	96.6	92.05	51.96
PP_3	89,870,702	84,589,878	94.12%	54.77%	96.46	91.78	53.61
IP_1	89,626,552	84,574,219	94.36%	58.56%	96.75	92.33	52.52
IP_2	89,357,710	84,599,747	94.68%	64.33%	96.88	92.6	50.84
IP_3	88,835,098	84,306,217	94.90%	56.81%	96.75	92.3	53.75
